# Prediagnostic circulating metabolites in female breast cancer cases with low and high mammographic breast density

**DOI:** 10.1038/s41598-021-92508-1

**Published:** 2021-06-22

**Authors:** Benedetta Bendinelli, Alessia Vignoli, Domenico Palli, Melania Assedi, Daniela Ambrogetti, Claudio Luchinat, Saverio Caini, Calogero Saieva, Paola Turano, Giovanna Masala

**Affiliations:** 1 Institute for Cancer Research, Prevention and Clinical Network (ISPRO), Via Cosimo il Vecchio 2, 50139 Florence, Italy; 2 Consorzio Interuniversitario Risonanze Magnetiche Di Metallo Proteine (CIRMMP), 50019 Sesto Fiorentino, Italy; 3 Magnetic Resonance Center (CERM), University of Florence, 50019 Sesto Fiorentino, Italy; 4 Department of Chemistry “Ugo Schiff”, University of Florence, 50019 Sesto Fiorentino, Italy

**Keywords:** Breast cancer, Biomarkers, Metabolic pathways

## Abstract

Mammographic breast density (MBD) is a strong independent risk factor for breast cancer (BC). We designed a matched case–case study in the EPIC Florence cohort, to evaluate possible associations between the pre-diagnostic metabolomic profile and the risk of BC in high- versus low-MBD women who developed BC during the follow-up. A case–case design with 100 low-MBD (MBD ≤ 25%) and 100 high-MDB BC cases (MBD > 50%) was performed. Matching variables included age, year and type of mammographic examination. ^1^H NMR metabolomic spectra were available for 87 complete case–case sets. The conditional logistic analyses showed an inverse association between serum levels of alanine, leucine, tyrosine, valine, lactic acid, pyruvic acid, triglycerides lipid main fraction and 11 VLDL lipid subfractions and high-MBD cases. Acetic acid was directly associated with high-MBD cases. In models adjusted for confounding variables, tyrosine remained inversely associated with high-MBD cases while 3 VLDL subfractions of free cholesterol emerged as directly associated with high-MBD cases. A pathway analysis showed that the “phenylalanine, tyrosine and tryptophan pathway” emerged and persisted after applying the FDR procedure. The supervised OPLS-DA analysis revealed a slight but significant separation between high- and low-MBD cases. This case–case study suggested a possible role for pre-diagnostic levels of tyrosine in modulating the risk of BC in high- versus low-MBD women. Moreover, some differences emerged in the pre-diagnostic concentration of other metabolites as well in the metabolomic fingerprints among the two groups of patients.

## Introduction

Mammographic breast density (MBD) extensively emerged as a strong risk factor for breast cancer (BC), regardless of any potential masking effect. A 3–5-fold increased BC risk was estimated among women in the highest quartiles of MBD in comparison with low-MBD women^1–4^. MBD is influenced by age, body mass index and by several hormonal, reproductive (such as parity, age at first birth, menopause and hormone use) and lifestyle factors which are also associated with BC risk^5–8^.

The well-known reduction of mammographic sensitivity in women with high MBD has been associated with a delayed diagnosis of BC in screened populations. Tumors detected in dense breast may thus, on average, have progressed to a higher stage, with worse prognostic characteristics at diagnosis^9,10^. Many studies examined the association between MBD, BC subtypes and a series of tumor characteristics at diagnosis^3,4,11–17^ and a recent review found evidence to support a positive relationship between high MBD, tumour size and lymph node positivity while no associations emerged between high MBD and subtype or receptor status^18^.

Metabolomics is the -omic science that deals with the characterization of the metabolome, i.e. the ensemble of low molecular weight molecules present in a biological specimen^19^. Since the metabolome is downstream of all the other omic products, it is the most affected by environmental factors, lifestyle, dietary habits, stress conditions, and drug treatments. As a consequence, metabolites can be thought as the most proximal reporters of any disease status or phenotype^20^, making metabolomics a technology with an enormous potential for the study and comprehension of the mechanisms of human health and diseases^21^.

Nuclear Magnetic Resonance spectroscopy (NMR) is suitable for the high-throughput, untargeted, metabolomics analysis of serum samples^22^ and provides a comprehensive picture of all metabolites present in the sample above the NMR detection limit (1 μM)^23^.

The applications of metabolomics in cancer research includes studies on disease etiology, biological mechanisms and metabolic pathways^24^. In particular, metabolomics in BC has been used to classify different cancer types, grades and stages, to identify markers for early diagnosis and prediction of prognosis and treatment outcomes^25–30^. Moreover, in prospective studies, metabolomics has been used to estimate the long-term risk of developing BC^31^. Being able to simultaneously quantify dozens of molecules in the same biological specimen, NMR could help finding new etiological pathways or biomarkers associated with BC in women with different levels of MBD.

We designed this study to investigate the differences in the metabolomic profiles of pre-diagnostic serum samples from a series of BC cases diagnosed in women with high- and low-MBD, through a matched case–case design. BC was not present at the time of blood sampling, but it was diagnosed during the follow up several years later. This design allows to evaluate for the first time, to the best of our knowledge, possible associations between the pre-diagnostic metabolomic profile and the risk of BC in women with high- versus low-MBD.

## Methods

### Study cohort

The European Prospective Investigation into Cancer and nutrition (EPIC) Florence cohort has been set up as a part of the EPIC European prospective study and enrolled (between 1993 and 1998) 10,083 clinically healthy women aged 35–64 years residing in the Florence area (Tuscany, Central Italy). All study participants signed an informed consent and gave permission to use the data collected during the study. The study was approved by the local Ethics Committee “Azienda Sanitaria Firenze”. All procedures performed were in accordance with the ethical standards of the institutional and national research committee and with the 1964 Helsinki declaration and its later amendments or comparable ethical standards.

At enrolment, weight, height, waist and hip circumferences were measured by trained nurses according to an international standard protocol. Data on frequency of consumption of 188 foods and drinks and usual portion size were obtained through a validated self-administered Food Frequency Questionnaire specifically developed to capture the Italian dietary habits. A standardized lifestyle questionnaire collected detailed information on reproductive history, smoking and alcohol drinking history, educational level, physical activity habits and medical history. Information on drug use including hormone replacement therapy (HRT) was also collected. Following a standardized protocol, a fasting blood sample was collected for every participant, processed, aliquoted and stored in the liquid nitrogen biological bank of the study, for long-term storage^32^.

The ascertainment of vital status was carried out through the linkage with the local town offices and the local Mortality Registry, thereby identifying the deceased subjects and the date and cause of death. Standardized follow-up procedures have been periodically implemented for the identification of cancer cases diagnosed after enrolment. The identification of BC cases (code C50 according to ICD-O-2 classification) was obtained through periodical linkage with the hospital discharge system and the Pathology Department registries^32^. At the 31/12/2015 follow up, 573 BC cases have been identified in the EPIC Florence cohort. Information on oestrogen receptor (ER) and progesterone receptor (PR) status was provided on the basis of pathology reports. Two categories (negative/positive) were considered according to well-established cut-off values (10% for both ER and PR)^33,34^.

In order to update the mammographic examination (ME) history of the EPIC female participants, we performed periodically a linkage with the mammographic archives of the population-based local mammographic screening (run by ISPRO, Florence) and of the MEs performed in a clinical setting at our Institution^35^. For each newly identified BC case we retrieved a negative ME performed at least one year before the BC diagnosis, if available, or otherwise the diagnostic ME. All MEs were revised by the study radiologist (DA) and classified according to the 4th Breast Imaging Reporting and Data System (BI-RADS) criteria: D1 < 25%, D2 = 25–50%, D3 = 51–75%, D4 > 75% of the area of the breast showing fibroglandular density. Overall, 481 out of the 573 identified BC cases have been classified according to the 4 BI-RADS categories^36^.

### Design of the nested case–case study

A case–case design was used to compare the pre-diagnostic metabolites' concentrations among low-MDB women who developed a BC (low-MBD cases) and among high-MDB women who also developed a BC (high-MBD cases).

A 1:1 case–case study was set up by selection of 100 high-MDB cases (MBD > 50%, BI-RADS = D3 or D4) and 100 low-MBD cases (MBD < 25%, BI-RADS = D1) matched by age at cohort entry (± 5 years), characteristics of the ME used to classify the MBD (analogical/digital; negative/diagnostic) and year of ME (before/after 31^st^ December, 1999). The population included in the present study consisted of all pairs that could be obtained through the above described procedure.

A total of 194 serum samples (97 complete case–case sets) were retrieved from the liquid nitrogen biological bank of the study and shipped to the study laboratory for the metabolomic profile examination. Metabolomic spectra were available for 174 serum samples corresponding to 87 complete case–case sets. Ten case–case sets were excluded because serum samples were of insufficient quality for metabolomics analysis (i.e. haemolyzed).

### Laboratory analysis

Serum samples were tested in the CERM laboratory (Centro di Risonanze Magnetiche) of the University of Florence, Italy. One-dimensional ^1^H NMR spectra were acquired at 310 K using a Bruker 600 MHz spectrometer (BrukerBioSpin) operating at 600.13 MHz proton Larmor frequency^37^. For each serum sample three standard 1D ^1^H NMR spectra namely CPMG (selective detection of low molecular weights metabolites), Diffusion-edited (selective detection of high molecular weights molecules), and NOESY (detection of all molecules present in concentrations above the detection limit) spectra were acquired. Samples were prepared and NMR spectra acquired following standard procedures^22^. Free induction decays were multiplied by an exponential function equivalent to a 0.3 Hz line-broadening factor before applying Fourier transform. Transformed spectra were automatically corrected for phase and baseline distortions and calibrated at the anomeric glucose signal at 5.24 ppm using TopSpin 3.2.

### Statistical analysis

Main baseline characteristics of BC cases were described separately for high- and low-MBD. Means, standard deviations and *p*-values from t test or Wilcoxon rank-sum tests were performed for continuous variables. Frequencies and Pearson’s chi-squared tests were performed for categorical variables.

Quantification of metabolites, lipid main fractions and subfractions was performed using the Bruker IVDr platform^38^. Completeness of measures and limits of quantification (LOQ) are shown in Supplementary Table 1. Values lower than the limit of quantification (LOQ) were imputed with half the LOQ. Metabolites with more than 20% of observation under the LOQ (n = 6) were excluded from the statistical analyses^39^.

Means of metabolite concentrations in high- and low-MBD cases were computed.

Metabolites, lipid main fractions and lipoprotein subfraction concentration values were log-transformed in order to normalize the distribution. Conditional logistic regression models were performed to estimate the association between metabolites, lipid main fractions and lipoprotein subfractions concentration and being a high-MBD case. Each single metabolite, lipid main fraction and lipoprotein subfraction (continuous, per standard deviation) was separately added to the model.

Additional models were performed in order to adjust for a set of potential confounding variables mainly related to MBD modulation (age at diagnosis, baseline menopausal status, number of full-term pregnancies, ER status, breastfeeding and baseline body mass index class,). Further models were also performed adjusting for waist/hip ratio, diabetes, hypertension and hyperlipidaemia. *p* values were adjusted for multiple testing using the false discovery rate (FDR) procedure with Benjamini–Hochberg correction at α = 0.05^40^. STATA 14.1 software was used for these analyses.

MetaboAnalyst^41,42^ was used to analyse the involved metabolic pathways related to the identified metabolites. The metabolic pathways analysis was conducted on the metabolites showing a significant association in conditioned logistic models, with the exclusion of lipid fractions that were not directly matchable with MetaboAnalyst. According to previous studies, only pathways with an impact > 0.2 were considered^43^.

To perform the multivariate analysis on the NMR spectra, each 1D spectrum in the range 0.2–10.00 ppm (thus the whole spectra, considering both assigned and unassigned metabolites) was segmented into 0.02 ppm chemical shift bins and the corresponding spectral areas were integrated using AMIX software (version 3.8.4, Bruker BioSpin). The region between 5.12 and 4.40 ppm containing the residual water signal was removed and the dimension of the system was reduced to 455 bins. The total spectral area was calculated on the remaining bins and total integral normalization was carried out prior to pattern recognition.

Unsupervised Principal Component Analysis (PCA) was used as first exploratory analysis to visualize the data and to discover possible outliers. Differences in the serum metabolomic fingerprints were then assessed using a supervised Orthogonal Partial Least Squares Discrimination Analysis (OPLS-DA) to cluster the groups of interest. In each OPLS-DA model the minimum number of latent variables that maximize model accuracy was retained (CPMG n = 7; NOESY n = 9; Diffusion n = 6). Accuracy, sensitivity and specificity for the OPLS-DA classifications were assessed by means of 100 cycles of a Monte Carlo cross-validation scheme (MCCV, R script in-house developed). Briefly, 90% of the data were randomly chosen at each iteration as a training set to build the model, the remaining 10% was tested and sensitivity, specificity and accuracy for the classification were assessed according to the standard definition. Significance of the classification results was assessed by means of a permutation test using 10^2^ permutations.

## Results

The MEs used to classify the MBD of BC cases were mostly analogical (75 of the 87 case–case sets; 86.2%), non diagnostic (73 sets; 83.9%) and performed after 31st December, 1999 (53 sets; 60.9%).

BC diagnosis occurred on average 8.6 and 8.2 years after blood sample collection in low- and high-MBD cases, respectively (*p* = 0.65). Mean age at diagnosis was significantly lower among high-MBD cases (62.9 and 59.8 years in low- and high-MBD cases, respectively, *p* = 0.002). High-MBD cases also showed a lower number of pregnancies and of breastfeeding months. Moreover low-MBD cases were mainly among post-menopausal women and among women with a higher body mass index (Table 1).

**Table 1:** Mean values and distribution of study participants according to the main characteristics for 87 case–case sets (EPIC Florence, low- vs high-MBD BC case–case study).

Variables	Low MBD cases N = 87	High MBD cases N = 87	*p* value
	Mean (SD) or N (%)	Mean (SD) or N (%)	
**Age at blood collection (years)**	54.3 (6.7)	51.6 (6.9)	0.01^j^
**Age at diagnosis (years)**	62.9 (6.4)	59.8 (6,8)	0.002^j^
**Length of follow-up from blood collection (years)**	8.6 (4.6)	8.2 (4.5)	0.65^k^
**Mean time between blood collection and mammographic examinations (years)**	6.1 (5.0)	6.4 (4.9)	0.78^j^
**Mean time between mammographic examinations and breast cancer (years)**	2.5 (2.8)	1.8 (2.6)	0.12^j^
**Diabetes**^a^			
No	84 (98.8)	85 (97.7)	
Yes	1 (1.2)	2 (2.3)	0.57^l^
**Hyperlipidaemia**^b^			
No	59 (70.2)	67 (77.0)	
Yes	25 (28.8)	20 (23.0)	0.32^l^
**Hypertension**^a^			
No	69 (81.2)	71 (81.6)	
Yes	16 (18.8)	16 (18.4)	0.94^l^
**Tumor characteristics**			
**Cancer size**^c^			
T1 (< 2 cm)	55 (71.4)	53 (71.6)	
T2 (2–5 cm)	9 (11.7)	10 (13.5)	
T3 (> 5 cm)	1 (1.3)	0 (0.0)	
T4 (any size, growing into the chest wall or skin)	1 (1.3)	0 (0.0)	
Tis (in situ)	11 (14.3)	11 (14.9)	0.73^ l^
**Lymph node status**^d^			
Negative	47 (77.0)	41 (66.1)	
Positive	14 (23.0)	21 (38.9)	0.60^ l^
**ER status**^e^			
Negative	6 (7.9)	13 (18,8)	
Positive	70 (92.1)	56 (81.2)	0.05^l^
**PR status**^e^			
Negative	35 (46.1)	27 (39.1)	
Positive	41 (53.9)	42 (60.9)	0.40^l^
**Reproductive history**			
**Age at first menstrual period (years)**	12.2 (1.6)	12.4 (1.4)	0.30^k^
**Contraceptive pill**^a^			
No	48 (56.5)	53 (60.9)	
Yes	37 (43.5)	34 (39.1)	0.55^l^
**Number of full-term pregnancies**			
0	8 (9.2)	20 (23.0)	
1	30 (34.5)	32 (36.8)	
2	33 (37.9)	33 (37.9)	
≥ 3	16 (18.4)	2 (2.3)	0.006^l^
**Age at first full-term pregnancy (years)**	26.0 (4.6)	26.9 (3.9)	0.24^j^
**Breastfeeding**			
No^g^	18 (20.7)	30 (34.5)	
Yes	69 (79.3)	57 (65.5)	0.04^l^
**Breastfeeding months** (126 breastfeeding women)	9.5 (6.6)	5.7 (3.3)	0.0001^j^
**Menopausal status at blood collection**^a^			
Premenopausal	21 (24.7)	34 (39.1)	
Postmenopausal	64 (75.3)	53 (60.9)	0.043^l^
**Menopausal status at mammographic examination**^f^			
Premenopausal	3 (3.6)	13 (15.5)	
Postmenopausal	81 (96.4)	71 (84.5)	0.009^l^
**Menopausal hormones use at blood collection**^**h**^			
No	42 (71.2)	34 (81.0)	
Yes	17 (28.8)	8 (19.0)	0.26^l^
**Menopausal hormones use at mammographic examination**^i^			
No	46 (93.9)	37 (90.2)	
Yes	3 (6.1)	4 (9.8)	0.52^l^
**Anthropometric measures**			
Height (cm)	160.5 (5.9)	161.0 (5.7)	0.56^j^
Weight (kg)	71.9 (11.4)	60.7 (8.4)	< 0.0001^j^
Body Mass Index (kg/m^2^)	27.9 (4.5)	23 (3.3)	< 0.0001^j^
Waist/hip ratio	0.80 (0.07)	0.76 (0.06)	< 0.0001^k^

^a^Missing = 2; ^b^missing = 3; ^c^missing = 23; ^d^missing = 51; ^e^missing = 29; ^f^missing = 6.

^g^No breastfeeding women or women with no full-term pregnancies.

^h^Postmenopausal women at blood collection = 117 (missing = 16).

^i^Postmenopausal women at mammographic examination = 152 (missing = 62).

^j^*p* value from t test.

^k^*p* value from Wilcoxon rank-sum test.

^l^*p* value from Pearson's chi-squared.

Fifteen metabolites (acetic acid, alanine, citric acid, creatine, creatinine, glucose, glutamine, glycine, histidine, isoleucine, lactic acid, leucine, pyruvic acid, tyrosine, valine), 7 lipid main fractions (triglycerides, cholesterol, LDL cholesterol, HDL cholesterol, APO A1, APO A2, APO B100) and 95 lipoprotein subfractions were quantified in the spectra. Mean concentrations according to low- and high-MBD are reported in Supplementary Table 2.

Logistic models conditioned on the matching variables showed that 6 out of the 15 metabolites were inversely associated with high-MBD BC cases: alanine (OR 0.59, 95%CI 0.42–0.83, *p* value 0.003); leucine (OR 0.71, 95%CI 0.52–0.98, *p* value 0.03); tyrosine (OR 0.59, 95%CI 0.42–0.82, *p* value 0.002); valine (OR 0.72, 95%CI 0.53–0.99, *p* value 0.04); lactic acid (OR 0.69, 95%CI 0.49–0.96, *p* value 0.03); pyruvic acid (OR 0.59, 95%CI 0.41–0.84, *p* value 0.003). Also the triglycerides lipid main fraction was inversely associated with high-MBD BC cases (OR 0.67, 95%CI 0.48–0.93, *p* value 0.02) as well as 11 VLDL subfractions of triglycerides, cholesterol, phospholipids and APO B (*p* value < 0.05). Acetic acid was directly associated with high-MBD BC cases (OR 1.67, 95%CI 1.19–2.35, *p* value 0.003). Overall, 7 metabolites (out of 15), 1 lipid fraction (out of 7) and 11 lipoprotein subfraction (out of 95) emerged as associated with high-MBD BC cases (Tables 2, 3).

**Table 2:** Association between metabolites concentration, lipid main fractions concentration and high-MBD BC cases, compared to low-MBD BC cases, in the 87 sets (EPIC Florence, low- vs high-MBD BC case–case study).

	Conditional logistic regression^a^		Adjusted conditional logistic regression^b^		
	Odds ratio (95% CI) for 1 SD	*p* value	Odds ratio (95% CI) for 1 SD	*p* value	*p* value FDR^c^
**Metabolite** (n = 15)					
Creatinine	0.89 (0.65–1.23)	0.490	0.82 (0.51–1.31)	0.408	NS
Alanine	0.59 (0.42–0.83)	0.003	0.61 (0.34–1.09)	0.095	NS
Creatine	0.78 (0.56–1.09)	0.149	0.73 (0.43–1.21)	0.222	NS
Glutamine	1.05 (0.79–1.38)	0.751	0.91 (0.59–1.39)	0.654	NS
Glycine	1.34 (0.98–1.85)	0.071	1.46 (0.91–2.34)	0.113	NS
Histidine	1.02 (0.76–1.37)	0.898	0.63 (0.36–1.08)	0.095	NS
Isoleucine	0.73 (0.52–1.01)	0.056	0.90 (0.56–1.45)	0.669	NS
Leucine	0.71 (0.52–0.98)	0.034	0.86 (0.53–1.39)	0.533	NS
Tyrosine	0.59 (0.42–0.82)	0.002	0.512 (0.27–0.94)	0.031	NS
Valine	0.72 (0.53–0.99)	0.044	0.89 (0.54–1.46)	0.633	NS
Acetic acid	1.67 (1.19–2.35)	0.003	1.47 (0.86–2.50)	0.158	NS
Citric acid	1.33 (0.97–1.83)	0.077	1.25 (0.80–1.94)	0.331	NS
Lactic acid	0.69 (0.49–0.96)	0.026	0.79 (0.46–1.35)	0.385	NS
Pyruvic acid	0.59 (0.41–0.84)	0.003	0.67 (0.39–1.16)	0.155	NS
Glucose	0.83 (0.60–1.14)	0.259	1.19 (0.71–1.97)	0.508	NS
**Lipid main fractions** (n = 7)					
Triglycerides	0.67 (0.48–0.93)	0.018	1.36 (0.77–2.39)	0.286	NS
Cholesterol	0.76 (0.54–1.08)	0.123	1.06 (0.67–1.68)	0.793	NS
LDL cholesterol	0.84 (0.61–1.15)	0.276	1.13 (0.71–1.79	0.608	NS
HDL cholesterol	1.23 (0.91–1.66)	0.173	0.81 (0.52–1.26)	0.348	NS
APO A1	1.05 (0.78–1.40)	0.756	0.85 (0.57–1.26)	0.419	NS
APO A2	0.92 (0.67–1.26)	0.607	0.88 (0.56–1.36)	0.554	NS
APO B100	0.72 (0.51–1.02)	0.068	1.23 (0.75–2.02)	0.405	NS

^a^Odds ratios per standard deviation (SD) increase in metabolite concentration conditioned on age at cohort entry (± 5 years), type of mammographic examination (analogical/digital; negative/diagnostic) and year of mammographic examination (before/after 2000). Single metabolites and lipid main fractions separately added to the regression model.

^b^Odds ratios per standard deviation (SD) increase in metabolite concentration conditioned on age at cohort entry (± 5 years), type of mammographic examination (analogical/digital; negative/diagnostic) and year of mammographic examination (before/after 2000) and adjusted for age at diagnosis, number of full-term pregnancies, breastfeeding (yes/no), menopausal status at baseline, ER status, body mass index at baseline. Single metabolites and lipid main fractions separately added to the regression model.

^c^*p* values adjusted for false discovery rate (FDR) at α = 0,05 with Benjamini–Hochberg correction.

**Table 3:** Association between lipoprotein subfractions concentration and high-MBD cases, compared to low-MBD BC cases, in the 87 sets (EPIC Florence, low- vs high-MBD BC case–case study).

	Conditional logistic regression^a^		Adjusted conditional logistic regression ^b^		
	Odds ratio (95%CI) for 1 SD	*p* value	Odds ratio (95%CI) for 1 SD	*p* value	*p* value FDR ^c^
LipoproteinMainFractionsTrigVLDL	0.65 (0.46–0.91)	0.014	1.43 (0.79–2.61)	0.238	NS
LipoproteinMainFractionsCholVLDL	0.68 (0.49–0.95)	0.025	1.57 (0.86–2.85)	0.141	NS
LipoprMainFractionsFreeCholVLDL	0.67 (0.47–0.93)	0.018	1.47 (0.82–2.64)	0.201	NS
LipoproteinMainFractionsPhosVLDL	0.66 (0.47–0.93)	0.018	1.50 (0.82–2.73)	0.186	NS
LipoproteinMainFractionsApoBVLDL	0.56 (0.33–0.96)	0.035	1.47 (0.85–2.53)	0.171	NS
SubfractionsTriglyceridesVLDL1	0.50 (0.31–0.80)	0.004	1.27 (0.63–2.56)	0.511	NS
SubfractionsTriglyceridesVLDL5	0.65 (0.46–0.92)	0.015	0.88 (0.53–1.47)	0.624	NS
SubfractionsCholesterolVLDL1	0.60 (0.42–0.86)	0.005	1.38 (0.75–2.52)	0.304	NS
SubfractionsCholesterolVLDL5	0.57 (0.33–0.98)	0.042	1.05 (0.51–2.16)	0.893	NS
SubfractionsFreeCholesterolVLDL2	1.04 (0.77–1.41)	0.790	2.05 (1.24–3.39)	0.005	NS
SubfractionsFreeCholesterolVLDL3	1.20 (0.87–1.66)	0.256	1.92 (1.08–3.41)	0.026	NS
SubfractionsFreeCholesterolVLDL4	1.04 (0.77–1.40)	0.818	1.72 (1.04–2.83)	0.035	NS
SubfractionsPhospholipidsVLDL1	0.55 (0.36–0.85)	0.006	1.49 (0.75–2.98)	0.259	NS
SubfractionsPhospholipidsVLDL5	0.51 (0.28–0.90)	0.021	1.02 (0.48–2.21)	0.951	NS

^a^Odds ratios per standard deviation (SD) increase in metabolite concentration conditioned on age at cohort entry (± 5 years), type of mammographic examination (analogical/digital; negative/diagnostic) and year of mammographic examination (before/after 2000). Single lipoprotein subfractions separately added to the regression model.

^b^Odds ratios per standard deviation (SD) increase in metabolite concentration conditioned on age at cohort entry (± 5 years), type of mammographic examination (analogical/digital; negative/diagnostic) and year of mammographic examination (before/after 2000) and adjusted for age at diagnosis, number of full-term pregnancies, breastfeeding (yes/no), menopausal status, ER status, body mass index. Single lipoprotein subfractions separately added to the regression model.

^c^*p* values adjusted for false discovery rate (FDR) at α = 0.05 with Benjamini–Hochberg correction.

In models adjusted for confounding variables (age at diagnosis, baseline menopausal status, number of full-term pregnancies, ER status, breastfeeding, baseline body mass index class) only tyrosine emerged as inversely associated with high-MBD cases (OR 0.51, 95%CI 0.27–0.94, *p* value 0.03) (Table 2), while 3 VLDL subfractions of free cholesterol showed a direct association with high-MBD cases (Table 3). Results did not change after further adjustment for waist/hip ratio, diabetes, hyperlipidaemia and hypertension (data not shown).

None of the examined molecules remained associated, in adjusted models, after controlling for multiple tests by FDR.

**Figure 1:**
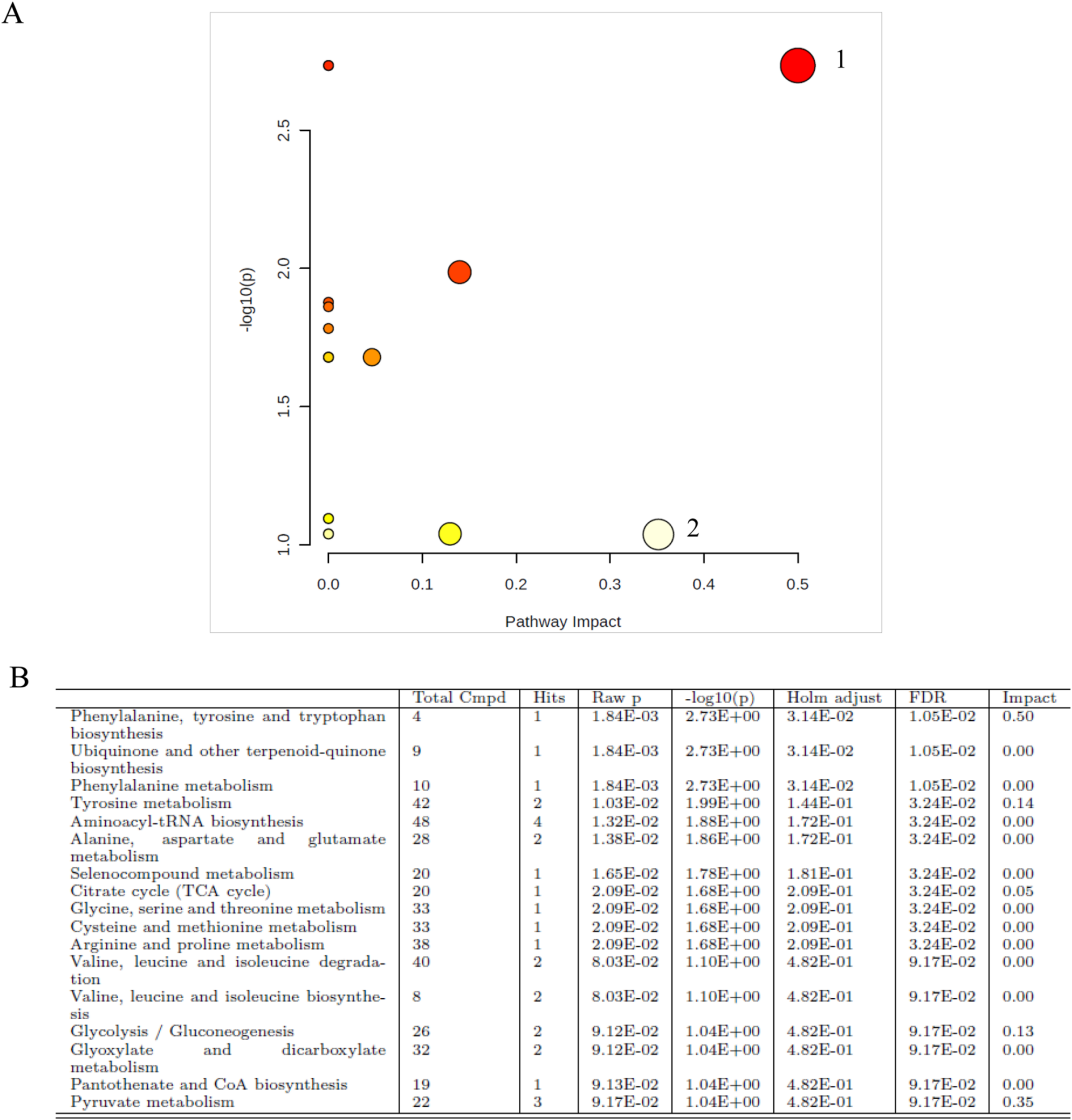
(**A**) Summary of pathways analysis using MetaboAnalyst (https://www.metaboanalyst.ca): (1) phenylalanine, tyrosine and tryptophan biosynthesis; (2) pyruvate metabolism. (**B**) Detailed results of pathway analysis. Total Cmpd is the total number of compounds in the pathway; Hits is the actually matched number of compounds from the user uploaded data; Raw *p* is the original *p* value calculated from the enrichment analysis; Holm is the *p* value adjusted by Holm-Bonferroni method; FDR is the *p* value adjusted using False Discovery Rate; Impact is the pathway impact value calculated from pathway topology analysis (EPIC Florence, low- vs high-MBD BC case–case study).

The results of the pathway analysis are presented graphically in Fig. 1. A total of 17 pathways were detected related to the 7 metabolites significantly associated with high-MBD cases in unadjusted logistic models conditioned on the matching variables. Two pathways emerged with an impact > 0.2. The first pathway was the “phenylalanine, tyrosine and tryptophan biosynthesis” (significant FDR adjusted *p* value), with 4 total compounds including 1 Hit corresponding to tyrosine. The second pathway was the “pyruvate metabolism” (non significant FDR adjusted *p* value). This pathway included 22 total compounds among which we had 3 Hits corresponding to pyruvic acid, lactic acid and acetic acid).

**Figure 2:**
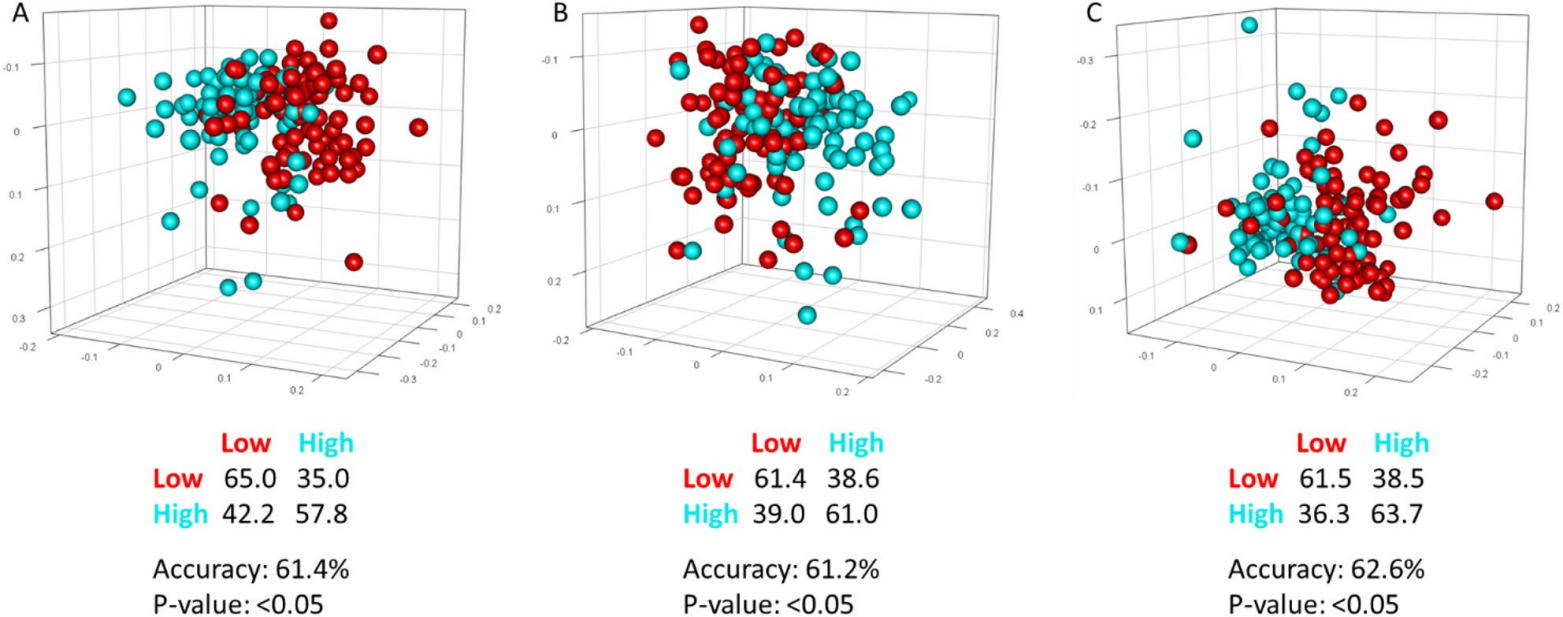
OPLS-DA score plot and confusion matrix for the comparison of low (red spheres) and high (cyan spheres) mammographic density breast cancer patients. The results are reported for the three kind of NMR spectra acquired: (**A**) NOESY, for low and high molecular mass molecules; (**B**) CPMG, for low molecular mass molecules; (**C**) Diffusion, for high molecular mass molecules. (EPIC Florence, low- vs high-MBD BC case–case study).

The PCA performed on the 87 case–case sets showed no outliers (Supplementary Fig. 1). Results of the supervised OPLS-DA for all the three types of NMR spectra (score plots: Fig. 2, loading plots: Supplementary Fig. 2) revealed a slight but significant separation between BC cases with high and low MBD (accuracy 61.2–62.6%, *p*-value < 0.05). The spectral regions that mainly contribute to the discrimination between high and low MBD women in the OPLS-DA models are those related to VLDL lipoproteins. Regarding low molecular weight metabolites, detected only in NOESY and CPMG spectra, the bins of alanine, valine, 3-hydroxybutyrate, pyruvate and lactate showed the highest discriminating power.

## Discussion

This study aimed to evaluate the possible differential role of individual pre-diagnostic metabolomic profiles in two matched series of BC cases identified in participants with low or high MBD. Pre-diagnostic serum samples from BC cases were examined in the frame of a case–case study nested in the EPIC Florence cohort. Since all study subjects developed BC, the investigated association is not to be interpreted as a BC risk assessment related to pre-diagnostic serum metabolites, but rather as an estimation of the possible differential effect of pre-diagnostic metabolomic profiles in the modulation of the risk to develop a BC in women with high *vs* low MBD.

As expected, a lower mean age at diagnosis emerged among high-MBD BC cases compared to their matched low-MBD BC cases. A reduction in MBD with aging has been extensively reported in literature^6^. The parameters associated with the reproductive history were also in line with the evidences reported in literature^5–7^. High-MBD cases occurred mainly in pre-menopause women and in women with a lower number of pregnancies.

As reported in literature^8^, in our study body mass index, body weight and waist and hip circumferences were significantly lower among high-MBD BC cases compared to low-MBD BC cases.

Results of the conditional logistic analyses showed an inverse association between serum levels of six metabolites and high-MBD BC cases, as well as for serum levels of the triglycerides lipid main fraction and 11 VLDL subfractions of triglycerides, cholesterol, phospholipids and APO B. One other metabolite was, on the other hand, directly associated with high-MBD BC cases. After adjustment for age at diagnosis, menopausal status, number of full-term pregnancies, breastfeeding, ER status and body mass index, tyrosine confirmed the significant inverse association with high-MBD BC cases and an association emerged between 3 VLDL subfractions of free cholesterol and high-MBD BC cases, although the reported associations lose significance after checking for multiple tests.

Alterations of amino acid levels in plasma or serum samples of breast cancer patients as compared with healthy controls were investigated by some studies with contradictory results^44^. Both tyrosine and alanine not only were found higher in BC patients, but their levels seem to be also influenced by the stage of the disease^45^. We observed higher levels of alanine and tyrosine in pre-diagnostic serum samples of low MDB cases in comparison to high MDB cases, although in our series no differences emerged in tumor characteristics of low- and high-MBD matched BCs.

In our study three free cholesterol VLDL subfractions showed a direct association with with high-MBD cases in adjusted models. Notably, our results remained significant in models adjusted for diabetes, hyperlipidaemia and hypertension. Obesity, overweight and dyslipidaemia are considered risk factors for BC, especially in postmenopausal women. However, the mechanisms in which they are involved, and therefore their role in BC development and growth, remain controversial probably due to different experimental settings^46,47^. Clinical studies and meta-analyses support a role for obesity, dietary fat intake and cholesterol in the onset and progression of BC, while some studies show that high cholesterol levels prior to diagnosis protect against the development of these tumors^46^. In a previous in vitro study LDL subfractions 1 and 5, VLDL, but not HDL, enhanced BC cell viability, increased the in vitro tumorigenesis, promoted BC cell migration and invasion and promoted angiogenic activity. However, only VLDL promoted metastasis in nude mice^47^. Moreover, in a previous in-vitro study, VLDL was associated with transport capacity of lipids to cancer cells to support breast cancer growth and development^48^.

Based on the pathway analysis the “phenylalanine, tyrosine and tryptophan biosynthesis” emerged as the most important metabolic process showing a differential expression among low- and high-MBD BC cases, that persisted after FDR testing. In a paper from Chen et al.^49^, tyrosine metabolism emerged as one of the most relevant dysfunctional pathways in aggressive cancer cell lines and an interaction between cancer related pathways and tyrosine metabolism was reported. Our pathway analyses also showed a possible role of the “pyruvate pathway”. Studies investigating the dysfunctional pathways that affect the progression of BC found “pyruvic metabolism” as one of the most closely involved. A series of differentially expressed genes such as ALDH2, ACACB and MDH1, contained in the “pyruvate metabolism” pathway, were down-regulated in BC samples^50,51^.

Finally, the untargeted supervised analyses performed on the serum metabolomic fingerprint revealed a slight but significant separation between BC cases diagnosed in women with high vs low MBD as assessed in a period preceding the diagnosis.

The main limitation of the current study is represented by the relatively modest sample size. Moreover, the smaller proportion of low-MBD women that were pre-menopausal and with a low BMI precluded the possibility to add menopausal status and BMI as criteria in the selection of the matched sets. However, menopausal status and BMI were considered as confounding variables in the adjusted models together with other variables strongly related to mammographic breast density, such as age at diagnosis, number of full-term pregnancies, breastfeeding and ER status. These adjusting variables strongly impacted the significance of the associations and, for some of the VLDL subfractions, also the direction of the associations. This strong impact was predictable since these characteristics are strongly related to mammographic breast density but also to the subjects metabolic/lipid profile. On the other hand, our study has several strengths. First of all the duration of the pre-diagnostic period between the sample collection and the BC diagnosis was very similar between the two matched series and sufficient to preclude any severe effect of BC on the metabolic profile of each individual study subject. Blood samples were collected and aliquoted according to standard operating procedures and have been stored in a dedicated liquid nitrogen biobank thus ensuring a good preservation of the serum samples.

To our knowledge, this is the first study to examine the differences in the metabolomic profile of pre-diagnostic samples of BC cases diagnosed in women with high- and low-MBD, through a matched case–case design.

To date, biomarkers identified as differentially expressed in blood of patients of certain types of cancer have been mainly used before cancer diagnosis for risk assessment and screening, at diagnosis for classification and staging and after diagnosis in monitoring treatments or cancer recurrence^52,53^. Few of these biomarkers have been tested rigorously in pre-diagnostic serum collected from asymptomatic subjects. The utility of available biomarkers for diagnosis of early BC is currently unknown^54^. Few studies have been conducted on large patient cohorts using pre-diagnostic blood samples to investigate possible associations between metabolic biomarkers and breast cancer risk. A prospective nested case–control study was set up in the SU.VI.MAX cohort, including 206 breast cancer cases diagnosed during a 13-year follow-up and 396 matched controls. Untargeted NMR metabolomic profiles were established from baseline plasma samples. Women characterized by higher plasma levels of valine, lysine, arginine, glutamine, creatine, creatinine and glucose, and lower plasma levels of lipoproteins, lipids, glycoproteins, acetone, glycerol-derived compounds and unsaturated lipids had a higher risk of developing breast cancer^55^. On the other hand, many clinical studies, recently included in a systematic review^56^, investigated the metabolomic biomarkers and the pathways related to BC diagnosis. Among 22 studies performed on plasma or serum samples, tyrosine was the most frequently mentioned metabolite related to BC diagnosis followed by other metabolites as alanine and glycine. Pathway analyses highlighted the role of alanine, aspartate and glutamate metabolism in BC development while pyruvate metabolism emerged among pathways with high, although not significant, impact^56^.

However only few studies, mainly validation studies, used pre-diagnostic blood samples to investigate potential cancer biomarkers. Despite recent progress in the detection of low level biomarkers in pre-diagnostic BC samples, the small samples size of studies and the background technical/biological noise still represent a challenge^57^. This reinforces the need to conduct larger exploratory studies in pre-diagnostic samples.

To conclude, in this case–case study aimed to identify metabolites differentially present in pre-diagnostic serum samples from high- or low-MBD women developing BC, a possible role for pre-diagnostic level of tyrosine in modulating the risk of BC in high- versus low-MBD women was suggested and some differences emerged in the pre-diagnostic metabolites concentration and in the distribution of the metabolomic fingerprints.

Larger studies are needed to expand these results in order to better understand the metabolic pathways leading to the development of BC in women with different levels of MBD.

## Supplementary Information

Supplementary Information.
