# Supplementary material for: Prediagnostic circulating metabolites in female breast cancer cases with low and high mammographic breast density
Source: Sci Rep. 2021 Jun 22;11:13025. doi: 10.1038/s41598-021-92508-1 (PMC8219761; doi:10.1038/s41598-021-92508-1)
Supplement: Supplementary file 1 — Supplementary Information. [file 41598_2021_92508_MOESM1_ESM.pdf]

Journal: Scientific Reports

Title:

Prediagnostic circulating metabolites in female breast cancer cases with low and high mammographic breast density.

Authors
Bendinelli B, Vignoli A, Palli D*, Assedi M, Ambrogetti D, Luchinat C, Caini S, Saieva C, Turano P, Masala G

Corresponding author
Domenico Palli, MD
postal address: Cancer Risk Factors and Lifestyle Epidemiology Unit, Institute for Cancer Research, Prevention and Clinical Network (ISPRO), Via Cosimo il Vecchio 2, 50139 Florence, Italy;
e-mail address: d.palli@ispro.toscana.it
telephone number: +39 055 416942 ext: 741

Supplementary Table 1. Data completeness for the different metabolites in the 174 low- and high MBD BC cases (87 complete sets). LOQ = limits of quantification.

**Metabolites**	**Results outside the measurable range**	
	**Samples, %**	**Criteria**
Creatinine	0	<LOQ (<0.01 mmol/L)
Alanine	0	<LOQ (<0.02 mmol/L)
Creatine	10.3	<LOQ (<0.01 mmol/L)
Glutamic acid	40.2	<LOQ (<0.05 mmol/L)
Glutamine	0	<LOQ (<0.02 mmol/L)
Glycine	0	<LOQ (<0.10 mmol/L)
Histidine	0	<LOQ (<0.02 mmol/L)
Isoleucine	2.3	<LOQ (<0.03 mmol/L)
Leucine	0	<LOQ (<0.01 mmol/L)
Threonine	38.5	<LOQ (<0.06 mmol/L)
Tyrosine	0.6	<LOQ (<0.03 mmol/L)
Valine	0	<LOQ (<0.03 mmol/L)
Acetic acid	0	<LOQ (<0.01 mmol/L)
Citric acid	0	<LOQ (<0.03 mmol/L)
Formic acid	24.7	<LOQ (<0.02 mmol/L)
Lactic acid	0	<LOQ (<0.03 mmol/L)
3-Hydroxybutyric acid	50.6	<LOQ (<0.02 mmol/L)
Acetone	21.8	<LOQ (<0.01 mmol/L)
Pyruvic acid	7.5	<LOQ (<0.03 mmol/L)
Glucose	0	<LOQ (<0.47 mmol/L)
Glycerol	71.8	<LOQ (<0.08 mmol/L)

Supplementary Table 2. Metabolites concentration among low- and high-MBD BC cases (EPIC Florence, low- vs high-MBD BC case-case study).

	**Low MBD cases N = 87**		**High MBD cases N = 87**	
	**Mean (SE)**	**95%CI**	**Mean (SE)**	**95%CI**
**Metabolites (mmol/dm^3)**				
Creatine	0.028 (0.002)	0.025 - 0.032	0.027 (0.002)	0.023 - 0.031
Alanine	0.455 (0.011)	0.434 - 0.476	0.407 (0.010)	0.388 - 0.426
Creatinine	0.076 (0.002)	0.073 - 0.080	0.075 (0.002)	0.071 - 0.078
Glutamic acid	0.055 (0.003)	0.049 - 0.061	0.051 (0.003)	0.045 - 0.058
Glutamine	0.752 (0.008)	0.737 - 0.768	0.759 (0.010)	0.739 - 0.779
Glycine	0.293 (0.008)	0.277 - 0.309	0.318 (0.010)	0.298 - 0.338
Histidine	0.086 (0.002)	0.083 - 0.089	0.086 (0.001)	0.083 - 0.088
Isoleucine	0.047 (0.003)	0.044 - 0.050	0.045 (0.001)	0.041 - 0.046
Leucine	0.095 (0.002)	0.091 - 0.100	0.088 (0.002)	0.084 - 0.093
Threonine	0.102 (0.009)	0.084 - 0.120	0.121 (0.010)	0.102 - 0.140
Tyrosine	0.061 (0.002)	0.059 - 0.064	0.054 (0.001)	0.051 - 0.056
Valine	0.229 (0.005)	0.219 - 0.239	0.214 (0.004)	0.206 - 0.223
Acetic acid	0.024 (0.002)	0.021 - 0.027	0.038 (0.008)	0.023 - 0.054
Citric acid	0.148 (0.005)	0.137 - 0.158	0.163 (0.007)	0.148 - 0.177
Formic acid	0.026 (0.001)	0.024 - 0.029	0.027 (0.001)	0.024 - 0.030
Lactic acid	2.070 (0.075)	1.922 - 2.219	1.857 (0.078)	1.703 - 2.011
3-Hydroxybutyric acid	0.026 (0.003)	0.020 - 0.0316	0.048 (0.006)	0.035 - 0.060
Acetone	0.013 (0.001)	0.011 - 0.015	0.017 (0.001)	0.015 - 0.019
Pyruvic acid	0.075 (0.003)	0.068 - 0.081	0.061 (0.004)	0.054 - 0.068
Glucose	4.708 (0.065)	4.579 - 4.837	4.598 (0.058)	4.482 - 4.713
Glycerol	0.120 (0.014)	0.093 - 0.147	0.110 (0.013)	0.084 - 0.136
**Lipid main fractions (mg/dL)**				
Triglycerides	124.3 (5.9)	112.7 - 135.9	108.7 (7.1)	94.6 - 122.7
Cholesterol	239.2 (4.7)	230.0 - 248.4	228.6 (4.8)	219.2 - 238.0
LDL cholesterol	132.5 (3.5)	125.7 - 139.3	126.2 (3.3)	119.7 - 132.7
HDL cholesterol	64.2 (1.3)	61.7 - 66.8	66.9 (1.4)	64.2 - 69.7
APO A1	164.9 (2.2)	160.7 - 169.2	166.1 (2.3)	161.6 - 170.6
APO A2	37.1 (0.5)	36.1 - 38.1	36.8 (0.6)	35.7 - 37.9
APO B100	100.7 (2.6)	95.5 - 105.9	93.7 (2.5)	88.9 - 98.6
**Lipoprotein subfractions (mg/dL)**				
LipoproteinMainFractionsTrigVLDL	74.0 (4.5)	65.1 - 82.8	61.6 (5.2)	51.3 - 71.9
LipoproteinMainFractionsTrigIDL	12.1 (0.9)	10.2 - 13.9	9.7 (1.2)	7.3 - 12.1
LipoproteinMainFractionsTrigLDL	21.4 (0.7)	20.1 - 22.8	20.4 (0.7)	19.1 - 21.8

LipoproteinMainFractionsTrigHDL	11.5 (0.4)	10.8 - 12.3	10.9 (0.4)	10.1 - 11.8
LipoproteinMainFractionsCholVLDL	22.6 (1.4)	19.8 - 25.4	18.6 (1.5)	15.5 - 21.6
LipoproteinMainFractionsCholIDL	15.2 (0.8)	13.5 - 16.9	12.8 (0.8)	11.2 - 14.5
LipoproteinMainFractionsCholLDL	132.5 (3.5)	125.7 - 139.3	126.2 (3.3)	119.7 - 132.7
LipoproteinMainFractionsCholHDL	64.2 (1.3)	61.7 - 66.8	66.9 (1.4)	64.2 - 69.7
LipoprMainFractionsFreeCholVLDL	10.1 (0.5)	9.0 - 11.2	8.5 (0.6)	7.3 - 9.7
LipoprMainFractionsFreeCholIDL	4.4 (0.2)	3.9 - 4.9	3.7 (0.2)	3.2 - 4.2
LipoprMainFractionsFreeCholLDL	39.4 (0.9)	37.5 - 41.2	38.4 (0.9)	36.6 - 40.1
LipoprMainFractionsFreeCholHDL	15.2 (0.4)	14.5 - 15.9	16.1 (0.4)	15.3 - 16.9
LipoproteinMainFractionsPhosVLDL	21.2 (1.2)	18.9 - 23.5	17.9 (1.2)	15.4 - 20.3
LipoproteinMainFractionsPhosIDL	8.3 (0.4)	7.5 - 9.0	7.1 (0.4)	6.3 - 8.0
LipoproteinMainFractionsPhosLDL	74.8 (1.7)	71.5 - 78.1	71.7 (1.6)	68.5 - 74.9
LipoproteinMainFractionsPhosHDL	90.7 (1.7)	87.4 - 94.0	93.3 (1.7)	90.0 - 96.6
LipoproteinMainFractionsApoA1HDL	166.6 (2.4)	162.0 - 171.3	168.8 (2.4)	164.0 - 173.6
LipoproteinMainFractionsApoA2HDL	37.4 (0.5)	36.4 - 38.4	37.0 (0.6)	35.8 - 38.1
LipoproteinMainFractionsApoBVLDL	8.5 (0.4)	7.6 - 9.4	7.3 (0.5)	6.3 - 8.2
LipoproteinMainFractionsApoBIDL	6.1 (0.3)	5.5 - 6.6	5.3 (0.3)	4.8 - 5.8
LipoproteinMainFractionsApoBLDL	84.4 (2.2)	80.1 - 88.7	79.6 (2.0)	75.6 - 83.7
SubfractionsTriglyceridesVLDL1	32.6 (2.5)	27.8 - 37.5	25.3 (2.9)	19.7 - 31.0
SubfractionsTriglyceridesVLDL2	12.0 (0.9)	10.2 - 13.8	10.2 (1.0)	8.2 - 12.3
SubfractionsTriglyceridesVLDL3	10.5 (0.8)	9.0 - 12.0	9.2 (0.9)	7.5 - 10.9
SubfractionsTriglyceridesVLDL4	8.9 (0.5)	8.0 - 9.8	7.8 (0.5)	6.8 - 8.9
SubfractionsTriglyceridesVLDL5	3.3 (0.1)	3.1 - 3.4	3.0 (0.1)	2.9 - 3.2
SubfractionsTriglyceridesLDL1	7.1 (0.2)	6.6 - 7.5	6.9 (0.2)	6.4 - 7.3
SubfractionsTriglyceridesLDL2	2.7 (0.1)	2.6 - 2.9	2.7 (0.1)	2.5 - 2.9
SubfractionsTriglyceridesLDL3	3.1 (0.1)	2.9 - 3.3	3.0 (0.1)	2.9 - 3.2
SubfractionsTriglyceridesLDL4	2.9 (0.1)	2.6 - 3.1	2.6 (0.1)	2.3 - 2.8
SubfractionsTriglyceridesLDL5	2.9 (0.2)	2.5 - 3.2	2.5 (0.2)	2.2 - 2.8
SubfractionsTriglyceridesLDL6	3.8 (0.2)	3.5 - 4.1	3.7 (0.2)	3.4 - 4.1
SubfractionsTriglyceridesHDL1	3.5 (0.2)	3.1 - 3.9	3.6 (0.2)	3.3 - 4.0
SubfractionsTriglyceridesHDL2	1.8 (0.1)	1.7 - 2.0	1.7 (0.1)	1.5 - 1.9
SubfractionsTriglyceridesHDL3	2.4 (0.1)	2.2 - 2.5	2.2 (0.1)	2.0 - 2.4
SubfractionsTriglyceridesHDL4	4.0 (0.1)	3.8 - 4.2	3.7 (0.1)	3.4 - 4.0
SubfractionsCholesterolVLDL1	7.2 (0.5)	6.2 - 8.2	5.6 (0.6)	4.5 - 6.7
SubfractionsCholesterolVLDL2	3.6 (0.3)	3.0 - 4.1	3.0 (0.3)	2.4 - 3.6
SubfractionsCholesterolVLDL3	3.8 (0.3)	3.2 - 4.4	3.1 (0.3)	2.5 - 3.7
SubfractionsCholesterolVLDL4	6.0 (0.4)	5.3 - 6.8	5.1 (0.4)	4.4 - 5.9
SubfractionsCholesterolVLDL5	1.9 (0.1)	1.7 - 2.0	1.7 (0.1)	1.5 - 1.8
SubfractionsCholesterolLDL1	31.2 (0.9)	29.5 - 32.9	30.3 (0.9)	28.4 - 32.1
SubfractionsCholesterolLDL2	19.8 (0.8)	18.2 - 21.4	21.3 (0.9)	19.4 - 23.1
SubfractionsCholesterolLDL3	20.0 (0.8)	18.5 - 21.5	20.0 (0.9)	18.4 - 21.7

SubfractionsCholesterolLDL4	20.0 (0.9)	18.3 - 21.7	17.5 (0.8)	15.9 - 19.2
SubfractionsCholesterolLDL5	18.9 (0.9)	17.1 - 20.6	16.0 (0.7)	14.6 - 17.4
SubfractionsCholesterolLDL6	22.6 (0.9)	20.8 - 24.4	21.0 (0.9)	19.2 - 22.9
SubfractionsCholesterolHDL1	18.4 (0.8)	16.8 - 20.1	20.9 (1.0)	18.8 - 22.9
SubfractionsCholesterolHDL2	9.3 (0.3)	8.7 - 9.8	9.7 (0.3)	9.2 - 10.2
SubfractionsCholesterolHDL3	12.1 (0.2)	11.6 - 12.5	12.1 (0.2)	11.7 - 12.6
SubfractionsCholesterolHDL4	23.1 (0.4)	22.3 - 23.8	22.9 (0.4)	22.1 - 23.7
SubfractionsFreeCholesteVLDL1	2.3 (0.2)	2.0 - 2.7	1.7 (0.2)	1.3 - 2.1
SubfractionsFreeCholesteVLDL2	1.3 (0.1)	1.1 - 1.5	1.0 (0.1)	0.8 - 1.3
SubfractionsFreeCholesteVLDL3	1.5 (0.1)	1.3 - 1.8	1.2 (0.1)	0.9 - 1.5
SubfractionsFreeCholesteVLDL4	2.5 (0.2)	2.2 - 2.8	2.0 (0.2)	1.7 - 2.3
SubfractionsFreeCholesteVLDL5	1.0 (0.0)	0.9 - 1.1	0.9 (0.0)	0.8 - 1.0
SubfractionsFreeCholesterLDL1	9.3 (0.3)	8.8 - 9.8	9.1 (0.3)	8.5 - 9.6
SubfractionsFreeCholesterLDL2	6.4 (0.2)	5.9 - 6.9	7.0 (0.3)	6.4 - 7.5
SubfractionsFreeCholesterLDL3	6.5 (0.2)	6.0 - 6.9	6.6 (0.2)	6.2 - 7.1
SubfractionsFreeCholesterLDL4	5.9 (0.2)	5.5 - 6.3	5.4 (0.2)	5.1 - 5.8
SubfractionsFreeCholesterLDL5	5.4 (0.2)	5.0 - 5.9	4.9 (0.2)	4.6 - 5.2
SubfractionsFreeCholesterLDL6	5.7 (0.2)	5.3 - 6.1	5.5 (0.2)	5.1 - 5.9
SubfractionsFreeCholesterHDL1	5.0 (0.2)	4.7 - 5.4	5.7 (0.2)	5.3 - 6.2
SubfractionsFreeCholesterHDL2	2.4 (0.1)	2.3 - 2.6	2.5 (0.1)	2.4 - 2.7
SubfractionsFreeCholesterHDL3	2.9 (0.1)	2.7 - 3.0	2.9 (0.1)	2.8 - 3.0
SubfractionsFreeCholesterHDL4	4.8 (0.1)	4.6 - 5.0	4.8 (0.1)	4.6 - 5.0
SubfractionsPhospholipidsVLDL1	5.4 (0.4)	4.5 - 6.2	4.1 (0.5)	3.2 - 5.1
SubfractionsPhospholipidsVLDL2	3.1 (0.2)	2.7 - 3.6	2.7 (0.3)	2.2 - 3.2
SubfractionsPhospholipidsVLDL3	3.5 (0.2)	3.0 - 4.0	3.0 (0.3)	2.4 - 3.5
SubfractionsPhospholipidsVLDL4	5.0 (0.3)	4.5 - 5.5	4.4 (0.3)	3.8 - 4.9
SubfractionsPhospholipidsVLDL5	2.2 (0.1)	2.0 - 2.3	1.9 (0.1)	1.7 - 2.1
SubfractionsPhospholipidsLDL1	17.5 (0.4)	16.6 - 18.3	17.0 (0.5)	16.1 - 18.0
SubfractionsPhospholipidsLDL2	11.2 (0.4)	10.4 - 12.0	11.9 (0.5)	11.0 - 12.8
SubfractionsPhospholipidsLDL3	11.2 (0.4)	10.5 - 12.0	11.2 (0.4)	10.4 - 12.1
SubfractionsPhospholipidsLDL4	11.2 (0.4)	10.3 - 12.0	9.9 (0.4)	9.0 - 10.7
SubfractionsPhospholipidsLDL5	10.3 (0.4)	9.4 - 11.2	8.9 (0.4)	8.2 - 9.6
SubfractionsPhospholipidsLDL6	12.9 (0.4)	12.0 - 13.8	12.2 (0.5)	11.3 - 13.2
SubfractionsPhospholipidsHDL1	23.1 (1.1)	21.0 - 25.2	26.0 (1.2)	23.6 - 28.4
SubfractionsPhospholipidsHDL2	14.8 (0.4)	14.0 - 15.6	15.1 (0.4)	14.4 - 15.9
SubfractionsPhospholipidsHDL3	19.6 (0.4)	18.9 - 20.3	19.5 (0.4)	18.8 - 20.2
SubfractionsPhospholipidsHDL4	32.5 (0.5)	31.6 - 33.4	32.0 (0.5)	31.0 - 33.0
SubfractionsApoA1HDL1	28.5 (1.5)	25.5 - 31.6	33.0 (1.8)	29.5 - 36.4
SubfractionsApoA1HDL2	21.7 (0.5)	20.7 - 22.7	22.3 (0.5)	21.3 - 23.3
SubfractionsApoA1HDL3	31.6 (0.6)	30.5 - 32.7	31.2 (0.6)	30.0 - 32.3
SubfractionsApoA1HDL4	85.6 (1.2)	83.3 - 87.9	83.5 (1.3)	81.0 - 86.1

SubfractionsApoBLDL1	16.3 (0.4)	15.4 - 17.1	15.9 (0.5)	14.9 - 16.8
SubfractionsApoBLDL2	11.2 (0.4)	10.4 - 11.9	11.8 (0.5)	10.9 - 12.7
SubfractionsApoBLDL3	11.8 (0.4)	11.0 - 12.5	11.7 (0.4)	10.8 - 12.5
SubfractionsApoBLDL4	12.9 (0.5)	11.8 - 13.9	11.2 (0.5)	10.2 - 12.2
SubfractionsApoBLDL5	13.4 (0.6)	12.2 - 14.6	11.5 (0.5)	10.4 - 12.5
SubfractionsApoBLDL6	18.7 (0.8)	17.2 - 20.3	17.5 (0.8)	15.9 - 19.0
SubfractionsApoA2HDL1	3.1 (0.2)	2.8 - 3.4	3.5 (0.2)	3.1 - 3.8
SubfractionsApoA2HDL2	4.1 (0.1)	3.9 - 4.3	4.1 (0.1)	3.9 - 4.3
SubfractionsApoA2HDL3	7.6 (0.2)	7.3 - 7.9	7.4 (0.2)	7.1 - 7.8
SubfractionsApoA2HDL4	22.4 (0.4)	21.6 - 23.2	21.8 (0.4)	20.9 - 22.6

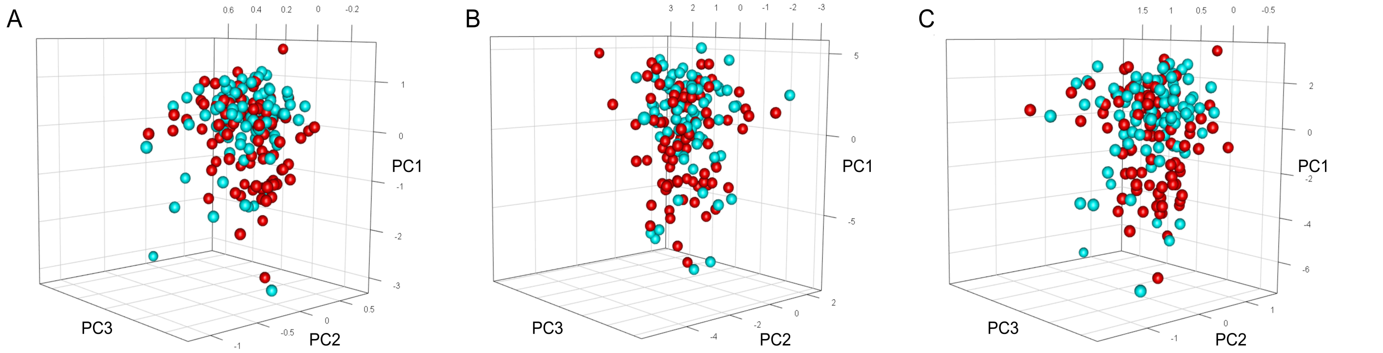

	PC1	PC2	PC3
Proportion of Variance	84.98%	9.40%	2.02%
Cumulative Proportion	84.98%	94.38%	96.40%

	PC1	PC2	PC3
Proportion of Variance	81.66%	7.88%	5.24%
Cumulative Proportion	81.66%	89.54%	94.78%

	PC1	PC2	PC3
Proportion of Variance	89.64%	5.50%	1.98%
Cumulative Proportion	89.64%	95.14%	97.12%

Supplementary Figure 1. PCA score plot and explained variance for the comparison of low- (red spheres) and high- (cyan spheres) MDB BC patients. The results are reported for the three kind of NMR spectra acquired, A) NOESY; B) CPMG; and C) Diffusion. (EPIC Florence, low- vs high-MBD BC case-case study).

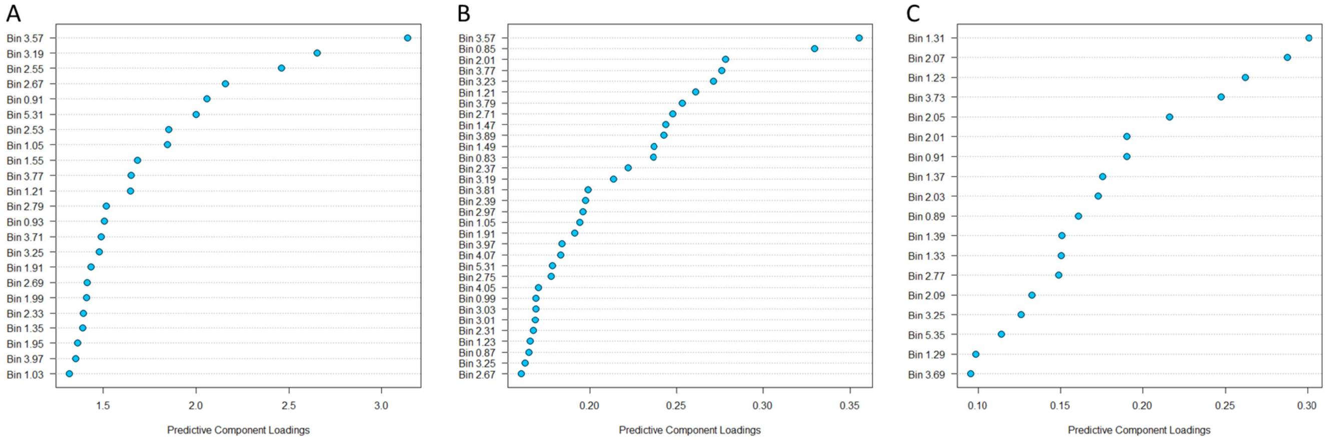

Supplementary Figure 2. Loading plots of the predictive component of the OPLS-DA calculated on the ^{1}H NMR spectra for the comparison of low- and high-MBD BC patients. The results are reported for the three kind of NMR spectra acquired, A) NOESY; B) CPMG; and C) Diffusion. (EPIC Florence, low- vs high-MBD BC case-case study). Only the bins that showed an intensity higher than two standard deviations of their averages are reported.
